# Possible role of transforming growth factor-β1 and vascular endothelial growth factor in Fabry disease nephropathy

**DOI:** 10.3892/ijmm.2012.1139

**Published:** 2012-09-24

**Authors:** MI HEE LEE, EUN NAM CHOI, YEO JIN JEON, SUNG-CHUL JUNG

**Affiliations:** Department of Biochemistry, School of Medicine, Ewha Womans University, Seoul 158-710, Republic of Korea

**Keywords:** Fabry disease, mouse, globotriaosylceramide, kidney, endothelial cell, transforming growth factor-β1, vascular endothelial growth factor

## Abstract

Fabry disease is a lysosomal storage disorder (LSD) caused by deficiency of α-galactosidase A (α-gal A), resulting in deposition of globotriaosylceramide (Gb3; also known as ceramide trihexoside) in the vascular endothelium of many organs. A gradual accumulation of Gb3 leads to cardiovascular, cerebrovascular and renal dysfunction. Endothelial cell dysfunction leads to renal complications, one of the main symptoms of Fabry disease. However, the pathological mechanisms by which endothelial dysfunction occurs in Fabry disease are poorly characterized. The purpose of this study was to investigate whether the expression of transforming growth factor-β1 (TGF-β1) and vascular endothelial growth factor (VEGF) is associated with the renal pathogenesis of Fabry disease. We found that the protein expression levels of renal thrombospondin-1 (TSP-1), TGF-β1 and VEGF were higher in the kidneys from Fabry mice compared to wild-type mice. The expression levels of VEGF receptor 2 (VEGFR2), fibroblast growth factor-2 (FGF-2) and phospho-p38 (P-p38) were also higher in the kidneys from Fabry mice compared with wild-type mice. Activities of cysteine aspartic acid protease (caspase)-6 and caspase-9 were higher in kidneys from Fabry than from the wild-type mice. These results suggest that overexpression of TGF-β1 and VEGF in the Fabry mouse kidney might contribute to Fabry disease nephropathy by inducing apoptosis. To test whether Gb3 accumulation can induce apoptosis, we incubated bovine aortic endothelial cells with Gb3 and found increased expression of TGF-β1, VEGFR2, VEGF, FGF-2 and P-p38. The combination of increased expression of TGF-β1 and VEGF caused by Gb3 accumulation may allow upregulation of FGF-2, VEGFR2 and P-p38 expression, and these changes may be associated with Fabry disease nephropathy by inducing apoptosis.

## Introduction

Fabry disease (OMIM 301500), also known as Anderson-Fabry disease, is a rare X-linked inherited lysosomal storage disorder (LSD) caused by deficiency of α-galactosidase A (α-gal A; EC 3.2.1.22) (1,2). A lack of lysosomal α-gal A enzyme causes accumulation of glycosphingolipids, mainly globotriaosylceramide (also known as Gb3, GL-3, or ceramide trihexoside) within the vascular endothelium in the brain, heart, liver, spleen, eyes, skin and kidney, leading to cerebrovascular, cardiac and renal complications (3). Renal insufficiency is observed in the final stage of life in a Fabry patient. However, structural changes in the kidney, including glomerulosclerosis and interstitial fibrosis, are observed even in young Fabry patients (4).

The Fabry mouse model has been established by disruption of the α-gal A-encoding gene (5). This knockout mouse model appears to be clinically normal, but ultrastructural analysis shows lipid inclusions in the liver and kidney. These pathophysiological changes in the α-gal A-knockout mouse are similar to those in patients with Fabry disease. An *in vitro* vascular cell model has been generated by culturing aortic endothelial cells from α-gal A-null mice (6). In the vascular endothelium of α-gal A-null mice, the deposition of Gb3 increases with age. Excessive Gb3 deposition in vascular endothelial cells is related to increased thrombosis and atherogenesis (7,8). The pathological mechanisms by which endothelial dysfunction occurs are poorly characterized. However, the pathogenic context of other kidney diseases including diabetes can provide clues for understanding the nephropathy of Fabry disease (9). Intracellular signaling proteins and growth factors such as insulin-like growth factors, transforming growth factor-β1 (TGF-β1) and vascular endothelial growth factor (VEGF) play a role in the development of diabetic nephropathy. These growth factors might also be related to the renal complication in patients with Fabry disease (9). TGF-β1 is known to play a key role in many diseases such as diabetes and renal disease (10,11). The TGF-β1 signaling pathway is mediated by mitogen-activated protein kinases (MAPKs), and TGF-β1 induces apoptosis in endothelial cells (12). VEGF is important in angiogenesis. VEGF binds to the VEGF receptors, fms-like tyrosine kinase [Flt-1, VEGF receptor 1 (VEGFR1)] and fetal liver kinase 1 (Flk-1, KDR, VEGFR2). VEGF expression is regulated by hypoxia, cytokines and growth factors (13). VEGF is expressed in glomerular podocytes and tubular epithelial cells.

Some investigators have proposed that the expression of TGF-β1 or VEGF is associated with Fabry nephropathy. Proteomic studies have demonstrated that circulating levels of VEGF and VEGFR2 are higher in young Fabry patients compared with controls (14,15). These observations suggest that TGF-β1 and VEGF expression is associated with Fabry nephropathy. We explored the roles of TGF-β1 and VEGF in the relationship between increased levels of Gb3 and endothelial dysfunction in renal pathogenesis in a mouse model of Fabry disease.

## Materials and methods

### Animals

Fabry mice were kindly provided by Dr Roscoe O. Brady (National Institutes of Health, Bethesda, MD, USA) and bred to produce sufficient numbers of mice for this study. To genotype each mouse, PCR was performed as described previously (5). Male mice were grouped into wild-type and hemizygous (Fabry) mice. Each group included a minimum of three animals. Sixteen-week-old mice were used for all experiments. All mice were provided with autoclaved water and diet *ad libitum*. All mice were treated in accordance with the Animal Care Guidelines of the School of Medicine, Ewha Womans University (Seoul, Korea). Fabry mice were treated with an injection of 1 mg Fabrazyme/kg (Genzyme, Cambridge, MA, USA) in saline through the tail vein.

### Cell culture

For *in vitro* study, bovine aortic endothelial cells (BAECs) were cultured in minimum essential medium (MEM) supplemented with 5% neonatal calf serum, 2 mM l-glutamine and penicillin/streptomycin (100 U/ml) at 37°C in a humidified 5% CO_2_ incubator. BAECs were used up to passages 7–9. All reagents were purchased from Gibco-BRL (Carlsbad, CA, USA).

### Western blot analysis

Proteins obtained from 50 mg of renal tissue were homogenized using Pro-Prep buffer (iNtRON Biotechnology, Inc., Seongnam-si, Korea) supplemented with phosphatase inhibitor cocktail solution (Dawinbio, Inc., Hanam-si, Korea). Tissue samples were incubated on ice for 30 min. Gb3-treated BAECs (80% confluent) were washed with ice-cold PBS and resuspended in PRO-PREP buffer supplemented with phosphatase inhibitor cocktail solution for 30 min on ice. Insoluble material was removed by centrifugation at 12,000 × g for 10 min at 4°C. Proteins (30–80 *μ*g) were separated on a 7.5–13.5% SDS-polyacrylamide gel and electrophoretically transferred to a polyvinylidene fluoride or nitrocellulose membrane (Millipore, Billerica, MA, USA). The membranes were blocked with 5% skim milk in Tris-buffered saline containing 0.1% Tween 20 (TBST) for 2 h at room temperature. The blots were then incubated with primary antibody overnight at 4°C. Antibodies used for western blot analysis were anti-thrombospondin (TSP)-1 monoclonal (NeoMarker, Oviedo, FL, USA); anti-cleaved cysteine aspatric acid protease (caspase)-6 polyclonal; anti-cleaved caspase-9 polyclonal; anti-cleaved caspase-12, phospho-p38 (P-p38) polyclonal; anti-VEGFR2 polyclonal (Cell Signaling Technology, Danvers, MA, USA); anti-fibroblast growth factor 2 (FGF-2) polyclonal (Abfrontier, Seoul, Korea); anti-TGF-β1 monoclonal (R&D Systems, Minneapolis, MN, USA); and anti-VEGF monoclonal (Santa Cruz Biotechnology, Inc., Santa Cruz, CA, USA) antibodies. The blots were washed with TBST three times for 5 min and then incubated with horseradish peroxidase-labeled secondary antibody for 1 h at room temperature. Goat anti-mouse IgG (1:2,500; Santa Cruz Biotechnology, Inc.) and goat anti-rabbit IgG (1:2,500; Santa Cruz Biotechnology, Inc.) were used as the secondary antibodies. After additional washes, the blots were detected by enhanced chemiluminescence using an ECL detection kit (GE Healthcare, Buckinghamshire, UK) according to the manufacturer’s instructions. The protein signals were visualized by exposing the membranes in a luminescent image analyzer (LAS-3000; Fujifilm, Tokyo, Japan). Each protein expression level was normalized to the expression of β-actin (Sigma-Aldrich, St. Louis, MO, USA). The results were quantified using Multi Gauge V3.0 software.

### Caspase-3/7 assay

Fifty micrograms of protein from kidney lysates (100 *μ*l) and caspase-3/7 substrate-containing solution (100 *μ*l) were mixed and incubated for 30 min to 1 h at 30°C in 96-well tissue culture test plates (SPL Life Sciences, Pocheon-si, Korea). The activity was measured on a Veritas Microplate Luminometer instrument (Promega Corporation, Madison, WI, USA). Caspase-3/7 substrate was purchased from Promega Corporation.

### Gb3 treatment

BAECs were seeded in 60 mm tissue culture plates (SPL Life Sciences) and were either untreated or treated with Gb3 (Matreya, Pleasant Gap, PA, USA) in complete MEM culture medium. The final Gb3 [dissolved in 100% dimethyl sulfoxide (DMSO; Sigma-Aldrich)] concentration of the treatment was 15 *μ*M.

### Data analysis and statistics

The values are presented as the mean ± SD or ± SE. Statistical comparisons between groups were performed using the Student’s t-test. P<0.05 was considered to indicate a statistically significant result.

## Results

### Protein expression of TSP-1, VEGF and TGF-β1 in kidneys of Fabry mice

**Figure 1:**
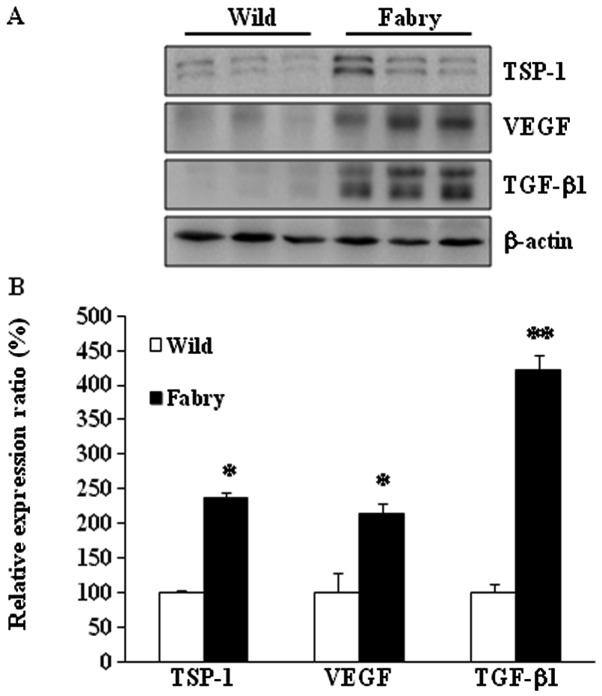
Protein expression levels of TSP-1, VEGF and TGF-β1 in the Fabry mouse kidney. (A) Results of western blot analysis using the antibodies described in Materials and methods. (B) Expression level of each protein was normalized to the internal control β-actin and represented as the expression ratio relative to that in wild-type mice. The values are expressed as the mean ± SD (n=3). ^*^P<0.05 and ^**^P<0.01, wild-type vs. Fabry mice.

The expression patterns of TSP-1, VEGF and TGF-β1 proteins were examined in the kidneys from Fabry and wild-type mice. As expected, TSP-1 protein expression was 234% higher in Fabry than in wild-type mice (Fig. 1) (P<0.05). VEGF protein expression was 214% higher in Fabry mice (P<0.05). TGF-β1 protein expression was 422% higher in Fabry than in wild-type mice (P<0.01). These results suggest that VEGF expression is influenced through another pathway associated with TGF-β1.

### Protein expression of VEGFR2, FGF-2 and P-p38 in kidneys of Fabry mice

**Figure 2:**
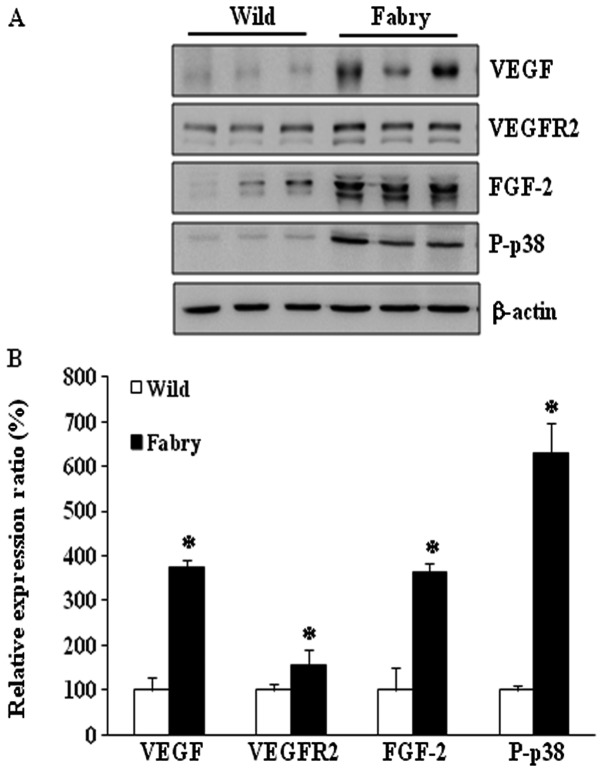
Protein expression levels of VEGF, VEGFR2, FGF-2 and P-p38 in the Fabry mouse kidney. (A) Results of western blot analysis using the antibodies described in Materials and methods. (B) The expression level of each protein was normalized to that of the internal control β-actin and is represented as the expression ratio relative to that in wild-type mice. The values are expressed as the mean ± SD (n=3). ^*^P<0.05, wild-type vs. Fabry mice.

Western blot analysis was performed to test the hypothesis that the combined overexpression of TGF-β1 and VEGF is associated with Fabry disease nephropathy. The protein expression of VEGFR2 was 157% higher in kidneys from Fabry than that in wild-type mice. FGF-2 expression was 365% higher in Fabry mice and P-p38 was 631% higher in Fabry than these levels in wild-type mice (Fig. 2). These results suggest that apoptosis signaling may be induced by the increased expression of TGF-β1 and VEGF in the kidney in the Fabry disease mouse model.

### Activation of caspases in the Fabry mouse kidney

**Figure 3:**
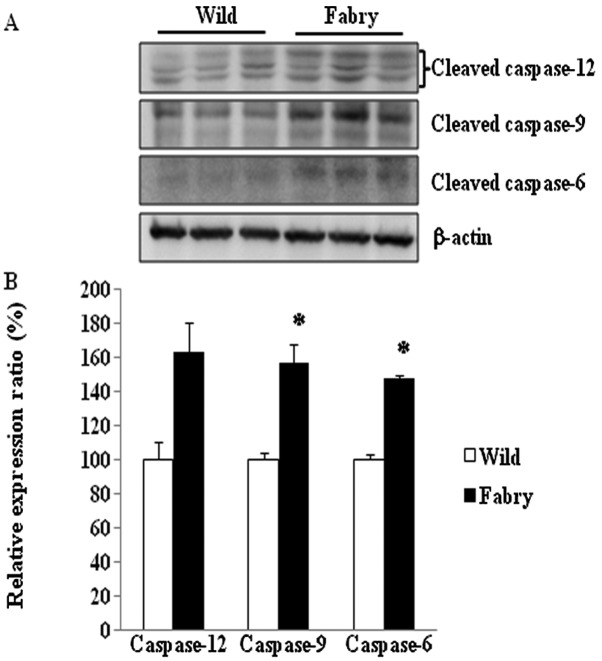
Protein expression levels of caspases in Fabry mouse kidney. Proteins from renal tissue were immunoblotted. (A) Results of western blot analysis using the antibodies described in Materials and methods. (B) The expression level of each protein was normalized to that of the internal control β-actin and is represented as the expression ratio relative to that in wild-type mice. The values are expressed as the mean ± SD (n=3). ^*^P<0.05, wild-type vs. Fabry mice.

**Figure 4:**
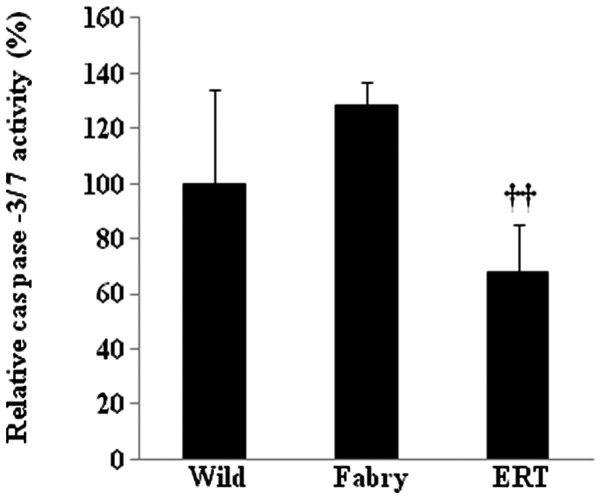
Measurement of caspase-3/7 activity in the Fabry mouse kidney. Fifty micrograms of renal tissue lysates was used to estimate caspase-3/7 activity (n=3). All data are represented as caspase-3/7 activity relative to that in the wild-type mice (%). ERT: Fabry mice were treated with an injection of α-gal A (1 mg Fabrazyme/kg) in saline through the tail vein. Tissues were sampled after 1 week. ^††^P<0.01, Fabry mice vs. ERT-treated Fabry mice.

The degree of activation of caspase-6, -9 and -12 was determined by measuring the levels of the cleaved forms of these caspases. The results shown in Fig. 3 indicate that the levels of cleaved caspase-6 (148%, P<0.05) and cleaved caspase-9 (157%, P<0.05) were significantly higher in Fabry mice than in wild-type mice. Cleaved caspase-12 level increased nonsignificantly (163%, P=0.06). To confirm that apoptosis was induced in the kidneys of Fabry mice, caspase-3/7 activity of key apoptotic proteins was measured using a colorimetric assay. The results shown in Fig. 4 indicate that caspase-3/7 activity was 128% higher in the kidney lysates from Fabry mice than in those from wild-type mice. In enzyme replacement therapy (ERT)-treated Fabry mice, activity of caspase-3/7 was 68% lower than that in the untreated Fabry mice (P<0.01).

### Effect of in vitro Gb3 treatment on BAECs from Fabry mice

**Figure 5:**
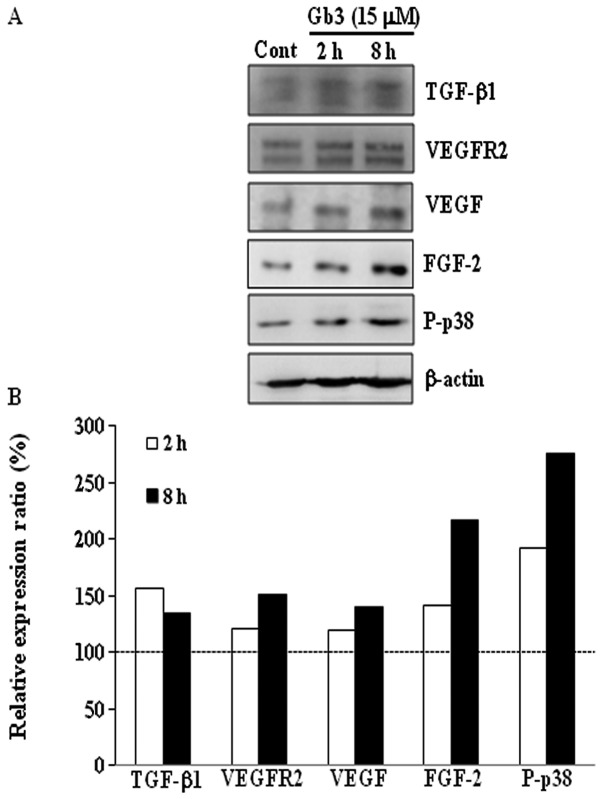
Protein expression levels of TGF-β1, VEGFR2, VEGF, FGF-2 and P-p38 in BAECs. BAECs were incubated with vehicle, DMSO alone (Cont) or 15 *μ*M Gb3 for 2 or 8 h. (A) Results of western blot analysis using the antibodies described in Materials and methods. (B) The expression level of each protein was normalized to that of the internal control β-actin and is represented as the expression ratio relative to that in the control BAECs. The dotted line represents the expression levels in the control BAECs.

To investigate whether Gb3 accumulation induces the expression of TGF-β1 and VEGF *in vitro* in BAECs in a manner similar to that observed in Fabry mice, BAECs were treated either with control (vehicle, DMSO alone) or 15 *μ*M Gb3 for 2 or 8 h (Fig. 5). The protein expression levels of TGF-β1, VEGFR2, VEGF, FGF-2 and P-p38 were higher in Gb3-treated BAECs than in control BAECs. Expression of these proteins was higher in BAECs treated with Gb3 for 8 h compared with 2 h.

## Discussion

Endothelial dysfunction related to excess Gb3 leads to renal complications in Fabry disease (3); however, the pathological mechanism responsible for the endothelial dysfunction caused by Gb3 accumulation is poorly understood. We hypothesized that growth factors such as TGF-β1 and VEGF, which play a role in the development of diabetic nephropathy, are upregulated in Gb3-accumulated endothelial cells and the Fabry mouse kidney, and that these factors play a crucial role in the development of the renal complications of Fabry disease (9).

Previously, we observed upregulation of lipocalin 2 (LCN2) and TSP-1 in the Fabry disease mouse and suggested that these molecules are candidate biomarker molecules for Fabry disease (16). To investigate further whether LCN2 induces TSP-1 expression and inhibits VEGF expression as reported previously (17), we examined the expression patterns of TSP-1 and VEGF in kidneys from Fabry and wild-type mice. We found higher TSP-1, VEGF and TGF-β1 expression levels in the kidneys from Fabry mice when compared with these levels in the wild-type mice (Fig. 1). These findings suggest that increased VEGF expression is associated with increased TGF-β1 expression.

TSP-1 is an extracellular matrix-remodeling glycoprotein and a crucial component of tissue remodeling and is associated with inhibition of angiogenesis (18). TSP-1 binds to multiple integrins and their receptors. The regulatory effects of TSP-1 are mediated by the interactions between TSP-1 and receptors (19). Zhang *et al*(20) reported that hepatocyte growth factor/scatter factor induces angiogenesis via TSP-1 downregulation and VEGF upregulation. In tumor cells, ectopic TSP-1 expression directly inhibits endothelial cell proliferation and survival, which promotes endothelial cell apoptosis. Thus, TSP-1 and VEGF can act as angiogenic regulators. In endothelial cells, TSP-1 induces apoptosis through activation of the Src-family tyrosine kinase (p59 Fyn), caspase-3-like proteases and p38. Subsequent activation of activator complex-1 (AP-1) leads to apoptosis (21,22). TSP-1 expression is increased in progressive renal disease and is associated with renal fibrosis (23) and TSP-1 stimulates TGF-β1 in diabetes (24). TSP-1 is a possible activator of TGF-β1 in kidney injury and can induce apoptosis of endothelial cells in many normal tissues (25,26). Consistent with previous reports, our data also showed that TSP-1 and TGF-β1 expression levels were higher in Fabry mice than in wild-type mice (Fig. 1).

VEGF increases vascular permeability, prevents apoptosis in endothelial cells (27,28) and induces apoptosis in cerebral endothelial cells after cell injury (29). Ferrari *et al*(30) suggested that TGF-β1 activates FGF-2 expression in endothelial cells, which then promotes VEGF production. They reported that TGF-β1 induces apoptosis via VEGF/VEGFR2-mediated phosphorylation of MAPK p38. Cross-talk between TGF-β1 and VEGF expression can alter the response of endothelial cells to apoptotic signals. Our results, shown in Figs. 1, 2 and 5, are consistent with these observations. Other investigators have also supported the hypothesis that TGF-β1 induces VEGF expression through MAPK (ERK1/2 and p38) activation (31) and that FGF-2 modulates VEGF expression in endothelial cells (32). Li *et al*(33) also suggested that VEGF-induced FGF-2 expression in injured endothelial cells leads to migration and proliferation of smooth muscle cells. VEGF stimulation results in TGF-β1-induced fibrosis in proximal tubular (NRK52E) cells (34). TGF-β1-induced epithelial cell apoptosis is associated with p38 (35). As discussed above, VEGF expression may not be downregulated by LCN2, but may be upregulated by TGF-β1.

We found that TGF-β1 and VEGF expression is increased in the Fabry mouse kidney and in Gb3-treated BAECs (Figs. 1 and 5), suggesting that TGF-β1 and VEGF upregulation may be associated with dysfunction of endothelial cells. Similarly, Sanchez-Nino *et al*(9) reported that the expression of TGF-β1, CD74 and extracellular matrix protein were increased by adding lyso-Gb3 (deacylated Gb3 form) to human podocytes, showing that TGF-β1 and CD74 are mediators of podocyte injury. CD74, the macrophage inhibitory factor receptor, is a potent receptor of kidney injury in diabetic nephropathy. Increased expression of TGF-β1 and/or VEGF in podocytes is associated with apoptosis or nephropathy (36,37). Our results are consistent with those from a rat nephropathy model in which TGF-β1 and VEGF expression increases (38). Therefore, we hypothesized that the combined overexpression of TGF-β1 and VEGF may be associated with Fabry disease nephropathy through induction of apoptosis.

We found significantly increased activities of caspase-6 and -9 in the Fabry mouse kidney and nonsignificant increases in the relative caspase-3/7 and -12 activities (Figs. 3 and 4). Caspase-9 and -12 are initiator caspases and caspase-3, -6, and -7 are effector caspases. In the final stage of the apoptosis pathway, effector caspases are activated by cleavage of cellular substrates (39). We found that apoptosis was induced in kidneys from Fabry mice when compared with wild-type mice. This apoptosis pathway might be related to the intrinsic pathway rather than to endoplasmic reticulum stress. Activity of caspase-3/7 in the kidney did not differ significantly between Fabry and wild-type mice but was lower in ERT-treated Fabry mice compared with Fabry mice. De Francesco *et al*(40) suggested that peripheral blood mononuclear cells (PBMC) from Fabry disease’s patients were in an apoptotic state, which correlated with the accumulation of Gb3. This suggests that Fabry nephropathy is associated at least partly with the intrinsic apoptotic pathway.

To investigate whether Gb3 treatment induces expression of TGF-β1 and VEGF in the endothelial cells, BAECs were treated either with control (DMSO alone) or with 15 *μ*M Gb3 for 2 or 8 h (Fig. 5). As expected, protein expression of TGF-β1 and VEGF increased in Gb3-treated BAECs. The combined expression of increased TGF-β1 and VEGF by Gb3 treatment may allow the upregulation of FGF-2, VEGFR2 and P-p38 expression (30). This finding is consistent with the increased protein expression observed in kidneys from Fabry mice.

Since apoptotic changes in renal biopsies from Fabry patients have not been demonstrated, apoptosis caused by the combined overexpression of TGF-β1 and VEGF cannot be the main pathogenic mechanism underlying the renal complications of Fabry disease (41,42). However, a greater apoptotic state has been observed in PBMC from Fabry patients (40,43), Gb3-induced apoptosis via TGF-β1 and VEGF overexpression may be associated with Fabry nephropathy.

In conclusion, we found upregulation of TGF-β1, VEGF, VEGFR2, FGF-2 and P-p38 expression in the Fabry mouse kidney and in Gb3-treated BAECs. Caspase-6 and -9 activation was also observed in the Fabry mouse kidney. The combined overexpression of TGF-β1 and VEGF by Gb3 may allow the upregulation of FGF-2, VEGFR2 and P-p38 expression. These results suggest that increased expression of these proteins is related to Fabry disease nephropathy through the induction of apoptosis.
